# Revealing spatiotemporal travel demand and community structure characteristics with taxi trip data: A case study of New York City

**DOI:** 10.1371/journal.pone.0259694

**Published:** 2021-11-09

**Authors:** Chen Xie, Dexin Yu, Xiaoyu Zheng, Zhuorui Wang, Zhongtai Jiang

**Affiliations:** 1 School of Transportation, Jilin University, Changchun, China; 2 Jilin Research Center for Intelligent Transportation System, Changchun, China; 3 Jilin Province Key Laboratory of Road Traffic, Changchun, China; Northeastern University (Shenyang China), CHINA

## Abstract

Urban traffic demand distribution is dynamic in both space and time. A thorough analysis of individuals’ travel patterns can effectively reflect the dynamics of a city. This study aims to develop an analytical framework to explore the spatiotemporal traffic demand and the characteristics of the community structure shaped by travel, which is analyzed empirically in New York City. It uses spatial statistics and graph-based approaches to quantify travel behaviors and generate previously unobtainable insights. Specifically, people primarily travel for commuting on weekdays and entertainment on weekends. On weekdays, people tend to arrive in the financial and commercial areas in the morning, and the functions of zones arrived in the evening are more diversified. While on weekends, people are more likely to arrive at parks and department stores during the daytime and theaters at night. These hotspots show positive spatial autocorrelation at a significance level of p = 0.001. In addition, the travel flow at different peak times form relatively stable community structures, we find interesting phenomena through the complex network theory: 1) Every community has a very small number of taxi zones (TZs) with a large number of passengers, and the weighted degree of TZs in the community follows power-law distribution; 2) As the importance of TZs increases, their interaction intensity within the community gradually increases, or increases and then decreases. In other words, the formation of a community is determined by the key TZs with numerous traffic demands, but these TZs may have limited connection with the community in which they are located. The proposed analytical framework and results provide practical insights for urban and transportation planning.

## 1 Introduction

The city is a large and complex system whose planning and transportation are closely linked to our daily lives. Quantifying the spatiotemporal patterns of residents’ travel flow can effectively reflect the dynamics of urban components [1]. Understanding the essence of it can provide us with some perspectives and insights for improving urban planning, transport efficiency, even energy conservation and emission reduction [2].

In the past, the acquisition of residents’ travel behaviors was mainly achieved through family travel surveys. The comprehensive information and its collection were labor-intensive and often with short application time and low accuracy [3, 4]. In recent years, there is increasing momentum in the analysis of urban travel through the data collected by travel-related digital sensors. The rapid popularity of high-granularity, multi-source data allows us to extract more accurate travel records and analyze the spatiotemporal mobility characteristics of the flow of people [5]. For example, many cities used smart cards to carry out statistical analyses [6, 7] and predict human travel behavior [8], employed taxi trajectory data to explore travel spatial distribution characteristics [9], and identified travel movement patterns through cell phone data [10]. Similar ideas have also been applied to mining spatiotemporal properties of emerging transportation modes, such as shared cars [11] and electric vehicles [12]. These studies generally found that humans congregate in different geographic locations over time. To further clarified influencing factors and aggregation patterns of travel behavior, many studies introduced spatial statistical models in cities and transport systems and carried out empirical arguments from multiple perspectives. These methods were mainly introduced into the spatial distribution statistical tests of the road network [13], urban bus systems [14], shared bicycles [15], parking [16], and even marine transport demand [17]. However, most of these studies were based on public transportation with fixed routes or short-distance travel, which often could not reflect the characteristics of crowd movement in random travel. The travel pattern of taxis and high-use private cars is more compatible with the city area but rarely appeared in existing studies.

Revealing the spatial pattern of urban traffic through network science does not have a long history. Here we present a brief review. Traditional research usually applied network analysis to street layouts based on urban topology [18]. However, the spatial interpretation of human activities by streets was controversial. Many studies were based solely on network topology methods, such as space syntax [13, 19], which ignored the flow of travel. In recent years, more and more studies divided the transportation system into multiple research units and used network science theory to analyze its flow characteristics and reveal its complex properties. Most research focused on discovering the community structure formed under different modes of transportation, such as revealed the multi-layer unified community structure of private cars, buses, and passengers [20], the evolution characteristics of the community structure of shared bicycle system [2, 15], community distribution of rail transit under different travel ratios [21]. In addition, some studies introduced complex network theory to calculate the clustering coefficient, path length, betweenness centrality in taxi travel networks [22] and confirmed the small-world property [23].

So far, the researches have focused on the illustration of spatial structures formed by different modes of transport, without revealing in depth the internal mechanism and the hidden characteristics of these structures. Past research carried out demonstrations based on the public system constrained by fixed stops, but the spatiotemporal characteristics of random travel behavior were not clear enough. This study fills these gaps and aims to develop a travel analysis framework with an empirical analysis in New York City. In this framework, kernel density estimation is used to discover the spatial hotspots where people depart and arrive. The global and local Moran’s I measure the spatial autocorrelation and cluster characteristics of traffic demand. Community detection extracts the network structures of travel. Based on network science, we analyze the weighted degree distribution of communities, the interaction property, and the similarities under different peak periods between the communities, which provide practical insights for urban and transportation planning.

The rest of the paper is organized as follows. In Section 2, we briefly describe the data sets and the division of analytical units. In Section 3, we introduce the overall logical framework and methodology of travel hotspots recognition, spatial autocorrelation, and community characteristics. In Section 4, we provide a case study based on New York City traffic network. In Section 5, we give a discussion of the results. In Section 6, we provide concluding remarks of the paper.

## 2 Study area and data

### 2.1 Study area

**Fig 1:**
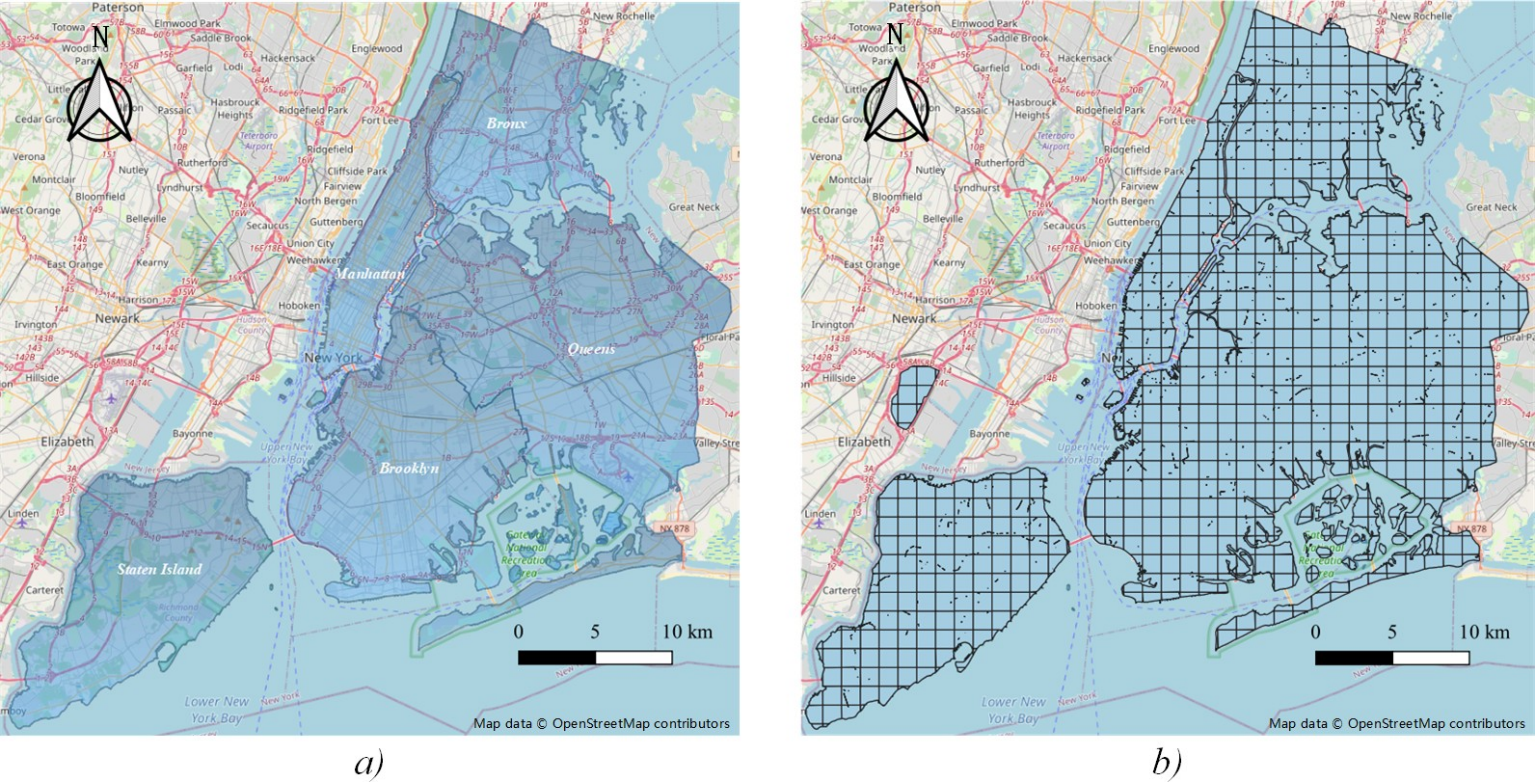
Layout and spatial division of study area.

New York City (NYC) is located in New York State, which is the most populous city in the United States. It is composed of five boroughs——Manhattan, Queens, Brooklyn, Bronx, and Staten Island (Fig 1). So far, taxicabs come in two varieties in NYC, yellow taxis can carry passengers in any city area, green taxis were launched in 2013 to solve the problem of lack of taxi services outside the downtown area. A full analysis of travel patterns at the city level requires the consideration of these two types. Based on taxi flow in the city, this study analyzes the spatiotemporal distribution of travel demand and specifically discusses the potential characteristics of the taxi community structure in NYC based on two varieties of taxi record data.

### 2.2 Data description and preprocessing

All the data employed in this study is the public data sets of the New York City Taxi and Limousine Commission (TLC) trip record data, including yellow, green taxis, and for-hire vehicle trip records in five boroughs of NYC, which contains the information of ID, pick-up and drop-off time stamp, coordinate, passenger count, trip distance and payment. After excluding extreme weather and holidays, we selected all taxi trip records from 2016.06.06 to 2016.6.12 to divide spatiotemporal units. The Isolated Forest algorithm [24] is used to clean data. There are a total of 2,005,334 travel records on weekdays and 775,836 on weekends after excluding the outliers and trips outside the study area.

### 2.3 Analytical unit division

**Fig 2:**
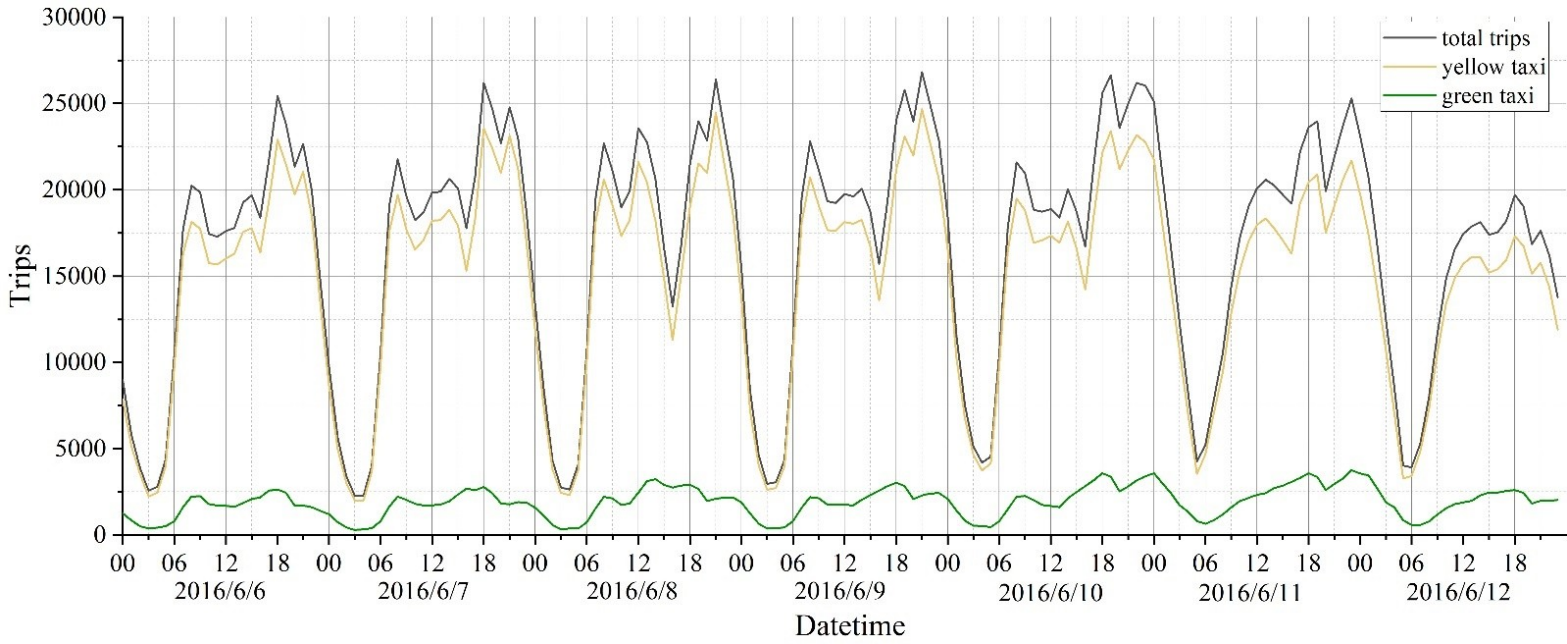
Time series of taxi flow.

An analysis of the trip time series is carried out in one-hour units (Fig 2). From 7 am on weekdays, the traffic flow increases strongly, lasting about until 10 am, and evening peak hours are from 5 pm to 8 pm. This feature is reflected in a similar pattern every day of a week. However, the traffic flow on weekends presents different times features. First, there is a small peak time between 12 pm and 3 pm, followed by a more prominent peak time between 5 pm and 8 pm. In addition, there is less traffic on Sunday than on Saturday. In summary, we select Tuesday and Saturday data that are representative and identify four peak periods for the following analysis Table 1.

**Table 1:** Time period definition.

Date	Period	
Tuesday (2016.6.7)	*peak1*	7 am~10 am
	*peak2*	5 pm~8 pm
Saturday (2016.6.11)	*peak3*	12 pm~3 pm
	*peak4*	5 pm~8 pm

Most studies selected grid units to group traffic sources and destinations, with sizes ranging from 500 meters to 2 kilometers to detect urban commuting traffic [25]. In our research, we select the size of 1 mile, which represents 25% of the overall trip distance. That is, 75% of the taxi passengers have a travel distance of more than 1 mile, which keeps a lot of information on the movement of taxis between different cells. Finally, 724 spatial analytical units are divided (Fig 1).

## 3 Methodology

### 3.1 Logic framework

**Fig 3:**
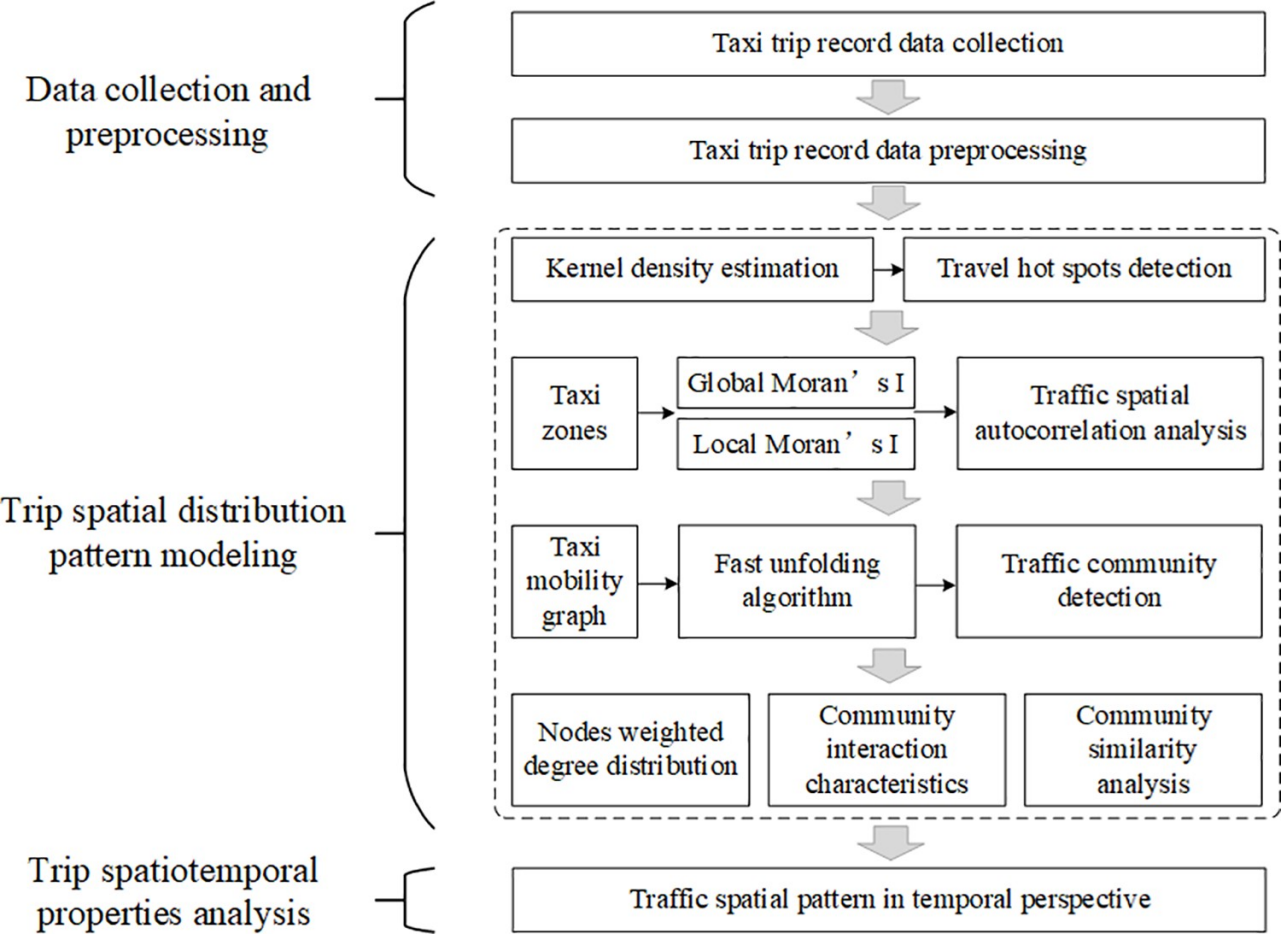
The logic framework of travel spatiotemporal analysis.

The overall logic framework of this research is shown in Fig 3, which is divided into 3 parts: data collection and preprocessing, trip spatial distribution pattern modeling, and trip spatiotemporal properties analysis.

### 3.2 Kernel density estimation

We use kernel density estimation (KDE) to find hotspots in different peak times. KDE is a non-parametric probability density estimation method, which has been used in spatial density analysis of point data [26–28]. If there is a space point *i* with the bandwidth *h*, then the kernel density estimator *D(x*,*y)* at the center point *(x*,*y)* is

$$D(x,y)=\frac{1}{nh^{2}}\sum_{i=1}^{n}K(\frac{d_{i}}{h})$$ (1)

where *d*_*i*_ is the distance between the space point and the center point. *K* is the kernel function. The quartic kernel described in Silverman’s work is used in this study [29], it is defined by

$$K(u)=\{\begin{array}{l} \frac{3}{\pi }{\left(1-u^{2}\right)}^{2},\;if\;0<d_{i}<h\\ 0,\;if\;d_{i}>h \end{array}$$ (2)

In hotspots recognition, the choice of bandwidth *h* needs to ensure that the OD points on roads are gathered around a plot. According to the area of the plots in this study, we set *h* = 300m, which can exploit the origin and destination (OD) hotspots based on trip record data from different periods in the urban transportation network effectively and perform spatial visualization analysis.

### 3.3 Spatial autocorrelation methods

#### 3.3.1 Global autocorrelation

Moran’s I index is introduced to explore the potential spatial autocorrelation of traffic demand. Global Moran’s I reveals the average degree of correlation between the data in the analysis space and the surrounding area. If there are *n* spatial units, the global Moran’s I of the traffic demand is

$$I=\frac{n}{S_{0}}\times \frac{\sum_{i=1}^{n}\sum_{j=1}^{n}w_{ij}(x_{i}-\bar{x})(x_{j}-\bar{x})}{\sum_{i=1}^{n}{\left(x_{i}-\bar{x}\right)}^{2}}$$ (3)

where $S_{0}=\sum_{i=1}^{n}\sum_{j=1}^{n}w_{ij}$. *x*_*i*_ and *x*_*j*_ are the value of traffic demand of the cell *i* and *j*, respectively. $\bar{x}$ is the average value of global traffic demand. *w*_*ij*_ is the spatial weight between *i* and *j*, it’s defined by *w*_*ij*_ = 1/*d*_*ij*_, *d*_*ij*_ represents the Euclidean distance between the two cells. The Z score is calculated as

$$Z=\frac{I-E(I)}{\sqrt{V(I)}}$$ (4)

where *E*(*I*) = −1/(*n*−1), *V*(*I*) = *E*(*I*^2^)−*E*(*I*)^2^, and *I*∈[–1,1], if *I* close to 1 with a high Z score indicates great positive spatial autocorrelation, while *I* close to -1 with low Z score indicates great negative spatial autocorrelation.

#### 3.3.2 Local autocorrelation

**Fig 4:**
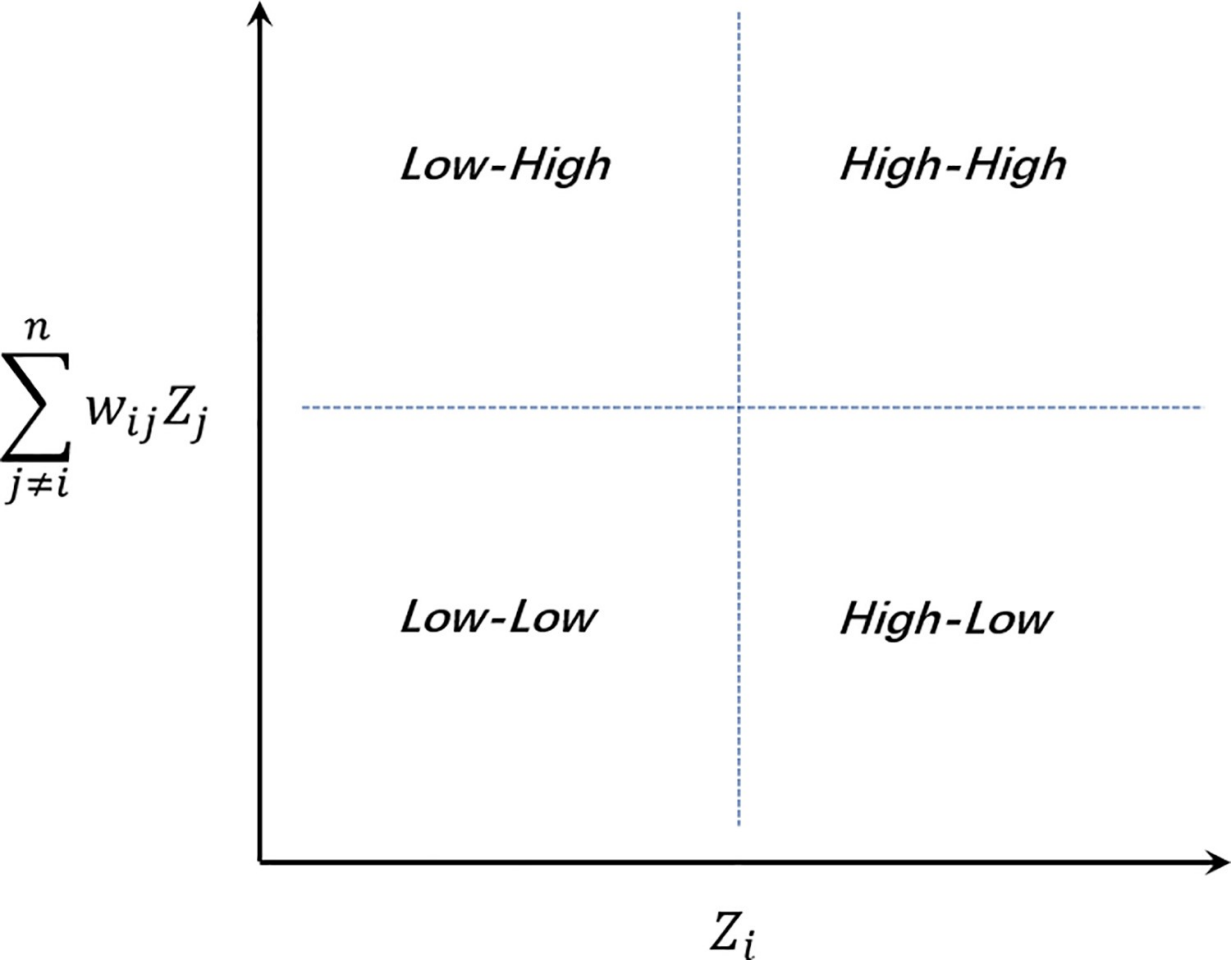
Local Moran’s I limit distribution.

The local Moran’s I (LISA) [30] calculates an index for each cell in the study area and identifies the specific location and type of correlation in the space. It is calculated by

$$I_{i}=\frac{Z_{i}}{S^{2}}\sum_{j\neq i}^{n}w_{ij}Z_{j}$$ (5)

where $Z_{i}=x_{i}-\bar{x},\;Z_{i}=x_{j}-\bar{x},\;S^{2}=\frac{1}{n}{\sum \left(x_{i}-\bar{x}\right)}^{2}$. The value of the local Moran’s I in unit *i* is determined jointly by two factors: 1) The similarity between unit *i* and the overall space (*Z*_*i*_). 2) The similarity between the surrounding areas of unit *i* and the overall space ($\sum_{j\neq i}^{n}w_{ij}Z_{j}$). It can be divided into four cases (Fig 4): (a) H-H: High values around neighbors with high values(cluster); (b) L-L: Low values around neighbors with low values(cluster); (c) H-L: High values around neighbors with low values(outlier); (d) L-H: Low values around neighbors with high values(outlier).

### 3.4 Traffic community detection

#### 3.4.1 Taxi mobility graph

Here, we construct a topological structure of multiple research units of the transportation network and map the travel information. An urban mobility system can be represented by a directed graph [15, 20], each cell (mentioned in 2.3) is regarded as a TZ. It can be defined by a directed graph *G* = {*V*,*E*} with nodes *V* = {*TZ*_*i*_|*i* = 1,2,…*n*}, where *n* is the number of TZs, and the trips between TZs as edges, denoted as *E* = *V*×*V* = {*e*_*ij*_}. For each trip *m* in the target period *T*, there is a trip information vector *Trip*_*m*_:

$$Trip_{m}=\{trip_{m}^{k}|trip_{m}^{k}=(t_{k},x_{m}^{tk},y_{m}^{tk}),\;k=1,2,\;t_{1}\in T\}$$ (6)

where *k* is an index, 1 for origin and 2 for destination. A trip *m* at time *t* includes origin and destination spatiotemporal information ${(t_{1},\;x}_{m}^{t1},\;y_{m}^{t1}),\;{(t_{2},\;x}_{m}^{t2},\;y_{m}^{t2})$, respectively. If a coordinate $\left(x_{m}^{tk},\;y_{m}^{tk}\right)$ falls inside the *TZ*_*i*_, then there is ${(x}_{m}^{tk},\;y_{m}^{tk})\in {TZ}_{i}$. Next, an OD matrix is represented by

$$OD=[\begin{array}{cccc} od_{11} & od_{12} & \cdots & od_{1n}\\ od_{21} & \ddots & & od_{2n}\\ \vdots & & & \vdots \\ od_{n1} & od_{n2} & \cdots & od_{nn} \end{array}]$$ (7)

Furthermore, we set the number of passengers as the edge weight *W* between origin and destination TZs, which denoted as *W* = *V*×*V* = {*w*_*ij*_|*w*_*ij*_ = *od*_*ij*_+*od*_*ji*_}.

#### 3.4.2 Fast unfolding algorithm

The community detection algorithm divides the network into sub-groups with tight internal connections between nodes, consisting primarily of three categories: division, cohesion, and optimization methods. The last depends on the solution of the objective functions without defining parameters artificially. Therefore, we choose the Fast unfolding algorithm [31] to reveal the community structures of the urban traffic network. It aims to maximize the modularity, for a weighted traffic network, the modularity is defined as

$$\begin{array}{l} Q=\frac{1}{2m}\sum_{i,j}\left[w_{ij}-\frac{k_{i}k_{j}}{2m}\right]\delta (C_{i},C_{j})\\ \delta (a,b)=\left\{ \begin{array}{l} 1\;when\;a==b\\ 0\;\mathrm{otherwise} \end{array}\right. \end{array}$$ (8)

where *m* is the sum of weights of the network, *w*_*ij*_ is the edge weight between *TZ*_*i*_ and *TZ*_*j*_. *k*_*i*_, *k*_*j*_ are the sum of weights of edges attached to *TZ*_*i*_ and *TZ*_*j*_, respectively. Where *k*_*i*_ = ∑_*j*_*w*_*ij*_, *k*_*j*_ = ∑_*i*_*w*_*ji*_. *C*_*i*_ is the community to where *TZ*_*i*_ is assigned. At the first stage, the algorithm calculates the modularity gain *ΔQ* by moving an isolated *TZ*_*i*_ into a community

$$\Delta Q=[\frac{\sum {}_{in}+2k_{i,in}}{2m}-{\left(\frac{\sum {}_{tot}+k_{i}}{2m}\right)}^{2}]-[\frac{\sum {}_{in}}{2m}-{\left(\frac{\sum {}_{tot}}{2m}\right)}^{2}-{\left(\frac{k_{i}}{2m}\right)}^{2}]$$ (9)

where ∑_*in*_ is the sum of edge weights within a community, while ∑_*tot*_ is the sum of edge weights of all nodes in the community. *k*_*i*,*in*_ denotes the sum of weights of edges originating from *TZ*_*i*_ to nodes in the community. Then the algorithm assigns node *TZ*_*i*_ to the community with the largest *ΔQ*. At the second stage, it treats the community generated in the previous step as a new node. Iteration until the modularity *Q* in the network is maximized, thus forming a network community structure with tight internal connections.

### 3.5 Community structure analysis

#### 3.5.1 Weighted degree and distribution

Based on the complex network theory, we specify several indicators for community structure analysis of TZs. For each peak period, we focus on quantifying the number of passengers between nodes rather than the number of trips. Therefore, the weighted degree [32–34] *deg*_*w*_(*TZ*_*i*_) is used to measure the importance of the node *TZ*_*i*_, which is defined by

$$\deg{\left(TZ_{i}\right)}_{in}=\sum_{i}e_{ij}$$ (10)

$$\deg{\left(TZ_{i}\right)}_{out}=\sum_{j}e_{ij}$$ (11)

$$\deg(TZ_{i})=\deg{\left(TZ_{i}\right)}_{in}+\deg{\left(TZ_{i}\right)}_{out}$$ (12)

$$\deg_{w}(TZ_{i})=\sum_{\deg(TZ_{i})}w_{ij}$$ (13)

where *deg*(*TZ*_*i*_)_*in*_ and *deg*(*TZ*_*i*_)_*out*_ are the in-degree and out-degree of node *TZ*_*i*_, respectively [34]. According to the definition of degree distribution [35], we introduce the idea of weighted degree distribution with the probability distribution of the weighted degree of TZs in the traffic network.

$$P_{k}(I)=\frac{N_{k}(I)}{N_{k}}$$ (14)

If the set of *Community* = {*C*_*k*_|*k* = 1,2,…*m*}, P_*k*_(*I*) is defined as the fraction of nodes in a community network *C*_*k*_ with a weighted degree within a numerical interval *I*. *N*_*k*_ is the number of nodes in the community *C*_*k*_, *N*_*k*_(*I*) is the number of nodes in the community *C*_*k*_ which nodes’ weighted degree falls in *I*.

#### 3.5.2 Interaction intensity

In the analysis of community interaction characteristics, we propose to use the edge weight and weighted degree of nodes [34] to quantify the intensity of node-community interactions. For a node *TZ*_*i*_, its interaction intensity *S*_*i*_ with community *C*_*k*_ is calculated by

$$S_{i,k}=\frac{\sum_{j}w_{ij}}{\deg_{w}(TZ_{i})}(TZ_{j}\in C_{k})$$ (15)

It can be understood as if *TZ*_*i*_ and *TZ*_*j*_ are in the same community, *S*_*i*,*k*_ measures the intensity of interaction with the community where *TZ*_*i*_ is located, otherwise, it measures the intensity of interaction with other communities. For each *TZ*_*i*_, there is $\sum_{k=1}^{m}S_{i,k}=1$.

#### 3.5.3 Similarity index

M. Yildirimoglu et al propose a similarity measurement of different transport network communities (bus network, passenger network, and car network) [20]. Based on this method, we introduce the similarity index of the traffic network community at different time periods. It is calculated by

$$\begin{array}{l} \sigma_{uv}=\frac{\sum_{i}\sum_{i\neq j}\alpha_{ij}^{u}\times \alpha_{ij}^{v}}{\min(\sum_{i}\sum_{i\neq j}\alpha_{ij}^{u},\sum_{i}\sum_{i\neq j}\alpha_{ij}^{v})}\\ \alpha_{ij}^{u}=\left\{ \begin{array}{l} 1\;\mathrm{if}\;i\;\mathrm{and}\;j\;\mathrm{are}\;\mathrm{in}\;\mathrm{the}\;\mathrm{same}\;\mathrm{community}\;\mathrm{in}\;\mathrm{time}\;\mathrm{period}\;u\\ 0\;\mathrm{otherwise} \end{array}\right. \end{array}$$ (16)

where *u* and *v* are different time periods, respectively. It measures the proportion of node pairs that are consistently allocated to the same community at different periods, where *σ*_*uv*_∈[0,1]. The numerator represents the number of OD pairs that are consistently assigned to the same community over the two periods. If an OD pair *od*_*ij*_ falls in the same community in the period *u* and *v*, there is $\sigma_{ij}^{u}\times \sigma_{ij}^{v}=1$.

## 4 Case study results

**Fig 5:**
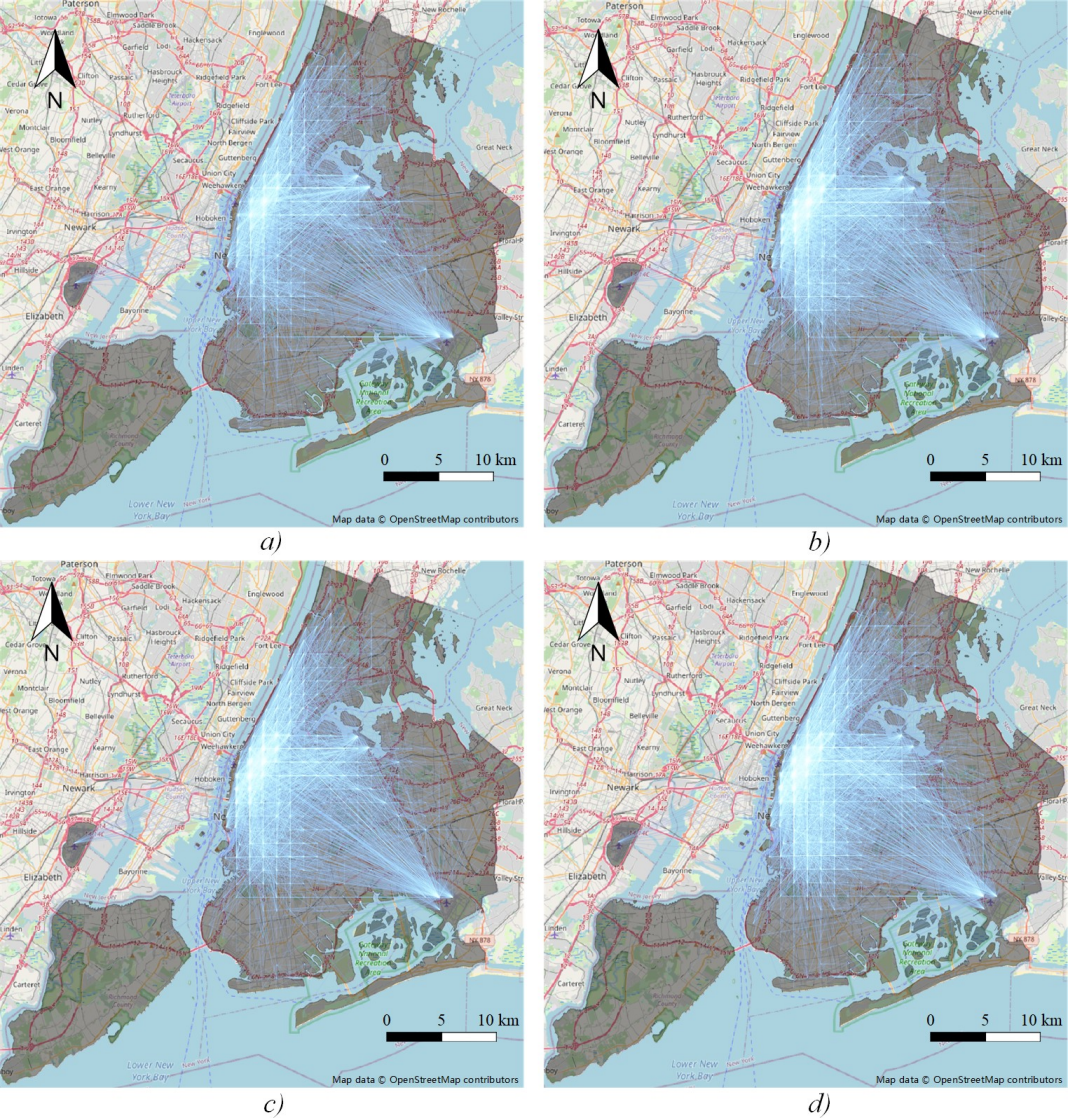
OD flow map. a) *peak1*; b) *peak2*; c) *peak3*; d) *peak4*.

The OD flow map of each time period is shown in Fig 5, with more obvious edges as the weight increases. Travel flows are mainly distributed in the surroundings of Manhattan and distant transportation hubs.

### 4.1 Hotspots detection

**Fig 6:**
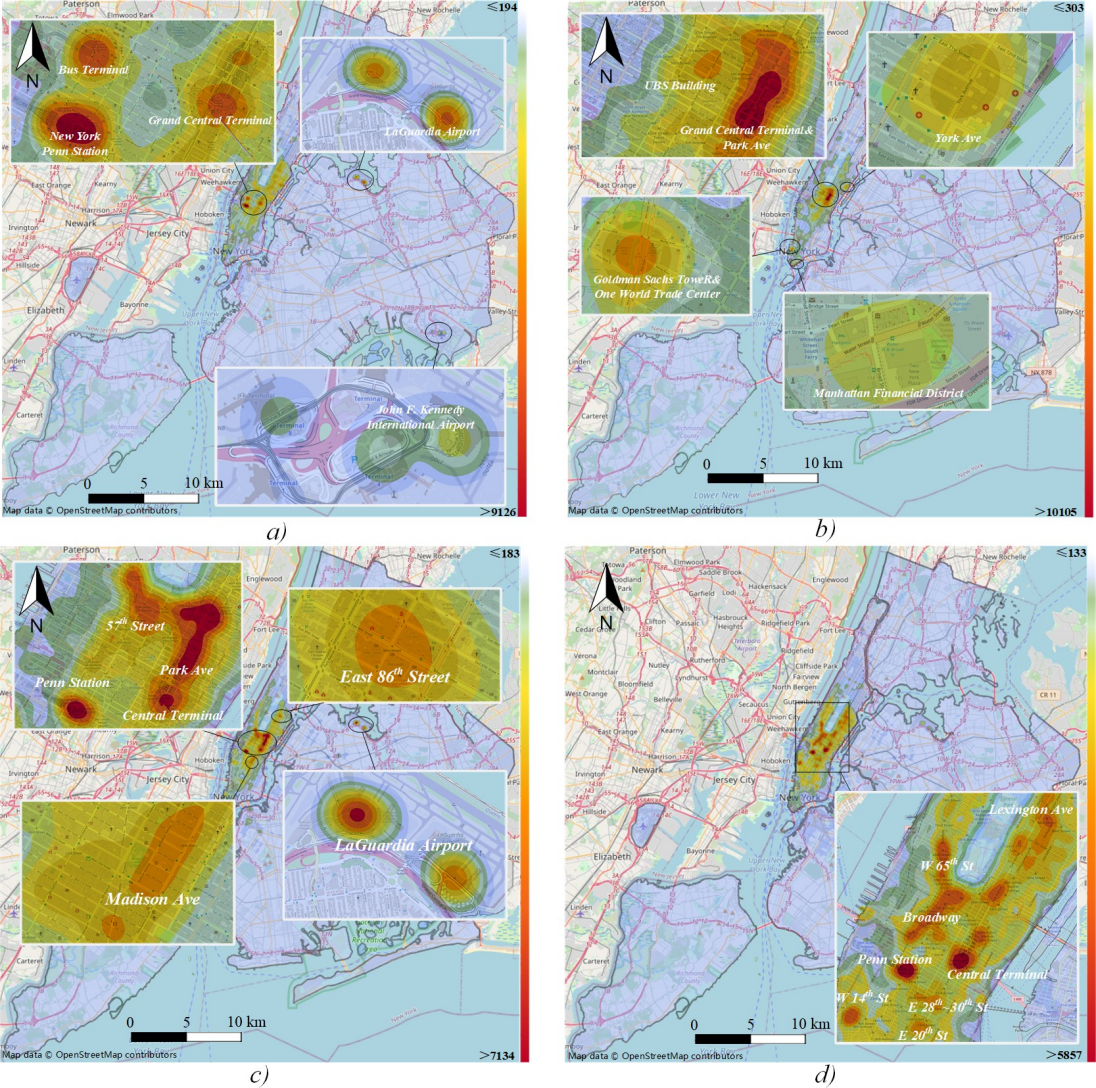
Hotspots of *peak1* and *peak2*. a) *peak1* origin; b) *peak1* destination; c) *peak2* origin; d) *peak2* destination.

**Fig 7:**
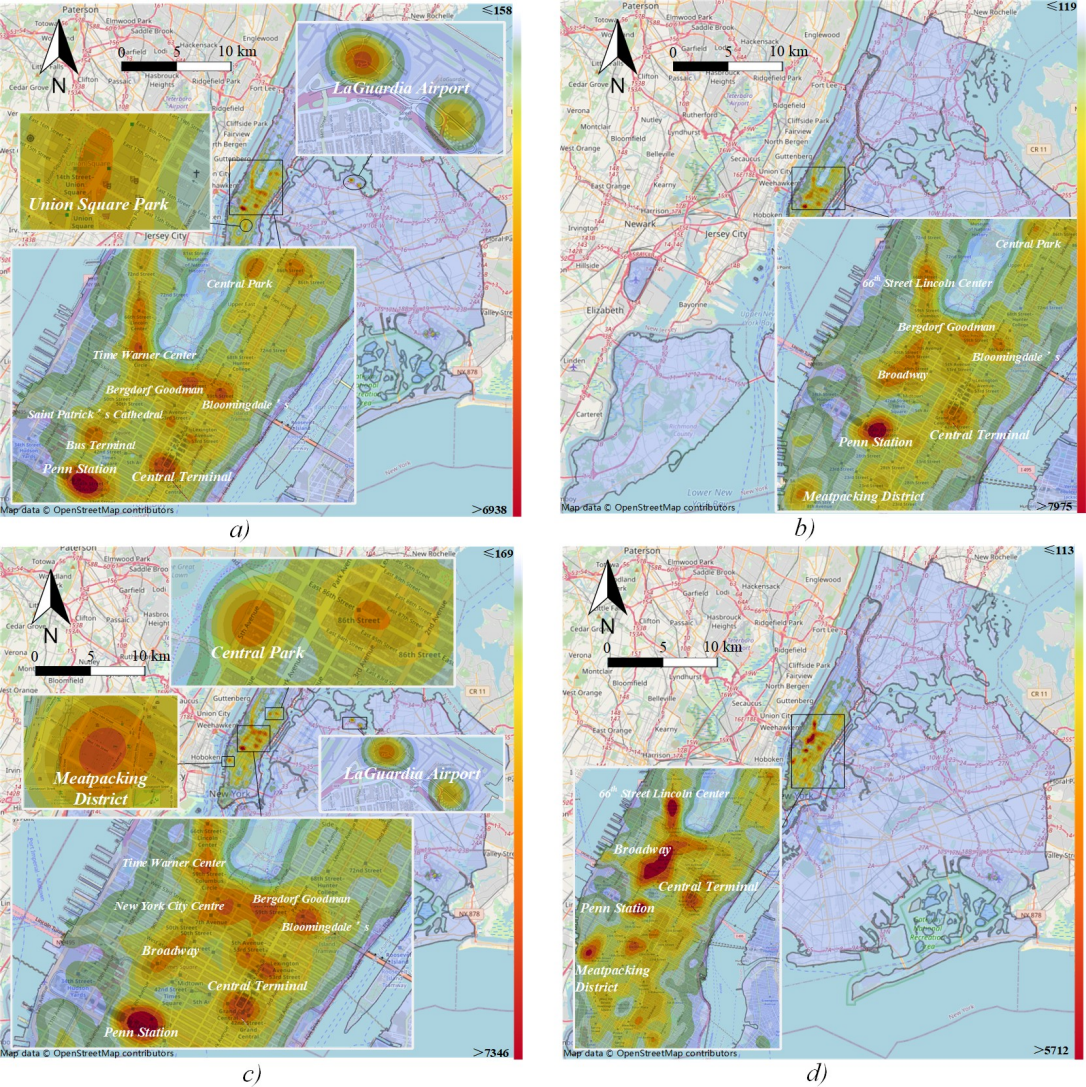
Hotspots of *peak3* and *peak4*. a) *peak3* origin; b) *peak3* destination; c) *peak4* origin; d) *peak4* destination.

The KDE method in ArcGIS is used to extract the hotspots from taxi OD points. A grid of 50m, *h* = 300 is selected for detailed visualization, and we use the natural break method to show the results (Figs 6 and 7). Manhattan is the most important financial and commercial district of NYC, with multiple transportation hubs. The high density of roads and the multiple land use functions make the most of trips originate from this area. On weekday, *peak1* origin demand (Fig 6A) is mainly concentrated in central Manhattan, widely distributed in the transportation hubs of the bus (Port Authority Bus Terminal), subway (Penn Station, Grand Central Terminal), and airport (LaGuardia Airport). Destination hotspots are concentrated in central and southern Manhattan, including the Grand Central Terminal and the surroundings of Park Avenue, with many commercial sites around it (Fig 6B). Moreover, the World Trade Center surrounding areas and the southern financial districts of Manhattan with light traffic concentrations (Fig 6B). At this time period, transportation is mainly used for commuting. We also find that the multiple medical sites on the Upper East Side and south of York Avenue have a slightly high concentration of traffic.

Taxi origin hotspots during *peak2* on weekday are mainly distributed at Penn Metro Station, Central Station, Park Avenue, 57th Street, East 86th Street, and LaGuardia Airport (Fig 6C). The southern part of the Park Avenue hotspot is a commercial area, while the northern part of Park Avenue, 57th Street, and Madison Avenue include a large number of catering facilities. Moreover, the East 86th Street hot zone, which includes banks and schools also shows a slight concentration. Destination hotspots are widely distributed, including land use for residential, catering, shopping, entertainment (Fig 6D). It can be inferred that some people do not return home directly after work, but engage in a series of other activities.

The taxi origin hotspots during *peak3* on weekends are also widely distributed in large public transportation hubs, like Penn Station, Grand Central Terminal, Port Authority Bus Terminal, and LaGuardia Airport (Fig 7A). Unlike weekdays, the department stores (Bergdorf Goodman, Bloomingdale), parks (Central Park, Union Square), and land use of worship (St. Patrick’s Cathedral) appear from the results. In addition to the places mentioned above, people also tend to get off in the surroundings of Broadway (Fig 7B), which located in many theaters. In general, people travel mainly for entertainment and socializing during this time.

The taxi origin hotspots of *peak4* and *peak3* are roughly the same. However, Meatpacking District is the unique origin hotspot of *peak4* (Fig 7C), where located American art museum, well-known clothing stores, and elevated parks. The destination hotspots in *peak4* still include department stores and parks but are more focused on Broadway (Fig 7D). It can be inferred that when traveling for entertainment, people tend to arrive at parks and department stores during the daytime and go to theaters at night.

### 4.2 Spatial autocorrelation analysis

Under the significance level of p = 0.001, the global Moran’s I and Z-score of each peak period are shown in Table 2.

**Table 2:** Results of global Moran’s I.

Type	Period	Global Moran’s I	Z-score
origin	*peak1*	0.729	15.580
	*peak2*	0.690	15.460
	*peak3*	0.737	16.041
	*peak4*	0.747	16.357
destination	*peak1*	0.598	16.137
	*peak2*	0.752	16. 052
	*peak3*	0.758	19.521
	*peak4*	0.779	20.755

**Fig 8:**
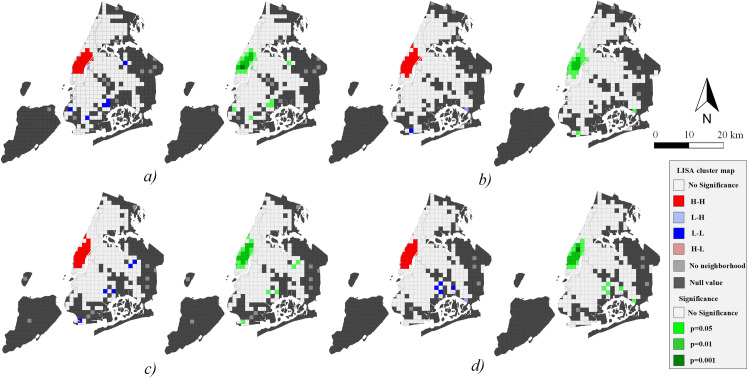
Spatial autocorrelation of origin demand. a) *peak1*; b) *peak2*; c) *peak3*; d) *peak4*.

**Fig 9:**
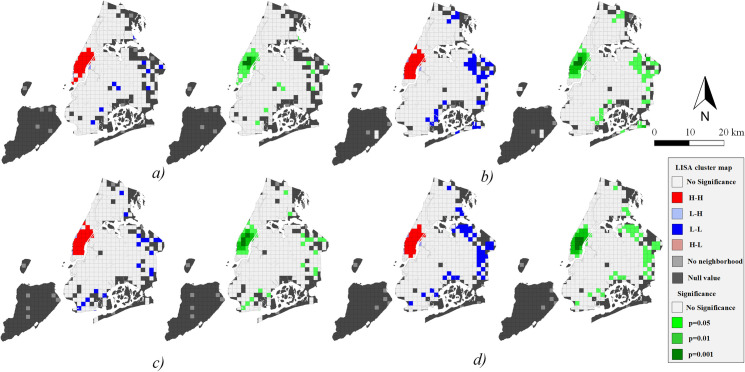
Spatial autocorrelation of destination demand. a) *peak1*; b) *peak2*; c) *peak3*; d) *peak4*.

The global Moran’s I in each period is positive and close to 1 with high Z scores, so traffic demand presents a significant clustering pattern during all peak times with a spatially positive correlation characteristic (Table 2). Furthermore, since the crowds are densely concentrated near Broadway during *peak4*, the spatial autocorrelation is higher than in other periods. The local Moran’s I pattern and statistical significance of trip volume with two subgraphs in each peak time are shown in Figs 8 and 9.

The traffic demand in the central area of Manhattan in NYC shows the characteristics of the H-H cluster in all periods. For origin demand during *peak1*, the most significant positive correlation area includes Penn Station with a level of p = 0.001 and then gradually decreases in surrounding areas (Fig 8A). While for the destination, the Grand Central Terminal and surrounding areas of Park Avenue have the highest level of significance, and also exist in southern Manhattan (Fig 9A). In other words, there is a tendency for passengers to travel south generally.

At the significance level of p = 0.001, *peak4* shows a significant positive correlation in the southern part of Central Park (origin demand), Union Park, Broadway, and transportation hubs (destination demand). We can see that taxis also have a southward flow trend (Figs 8D and 9D). In addition, traffic demand during every peak period has L-H clustering in the Queens-Midtown tunnel area, while passenger arrivals show L-L clustering in the east of NYC (Fig 9). The spatial distribution of taxi destinations is wider than origin. It can be inferred that taxis tend to carry passengers in the central area of Manhattan and drive them to the far peripheries.

### 4.3 Community structure analysis

**Fig 10:**
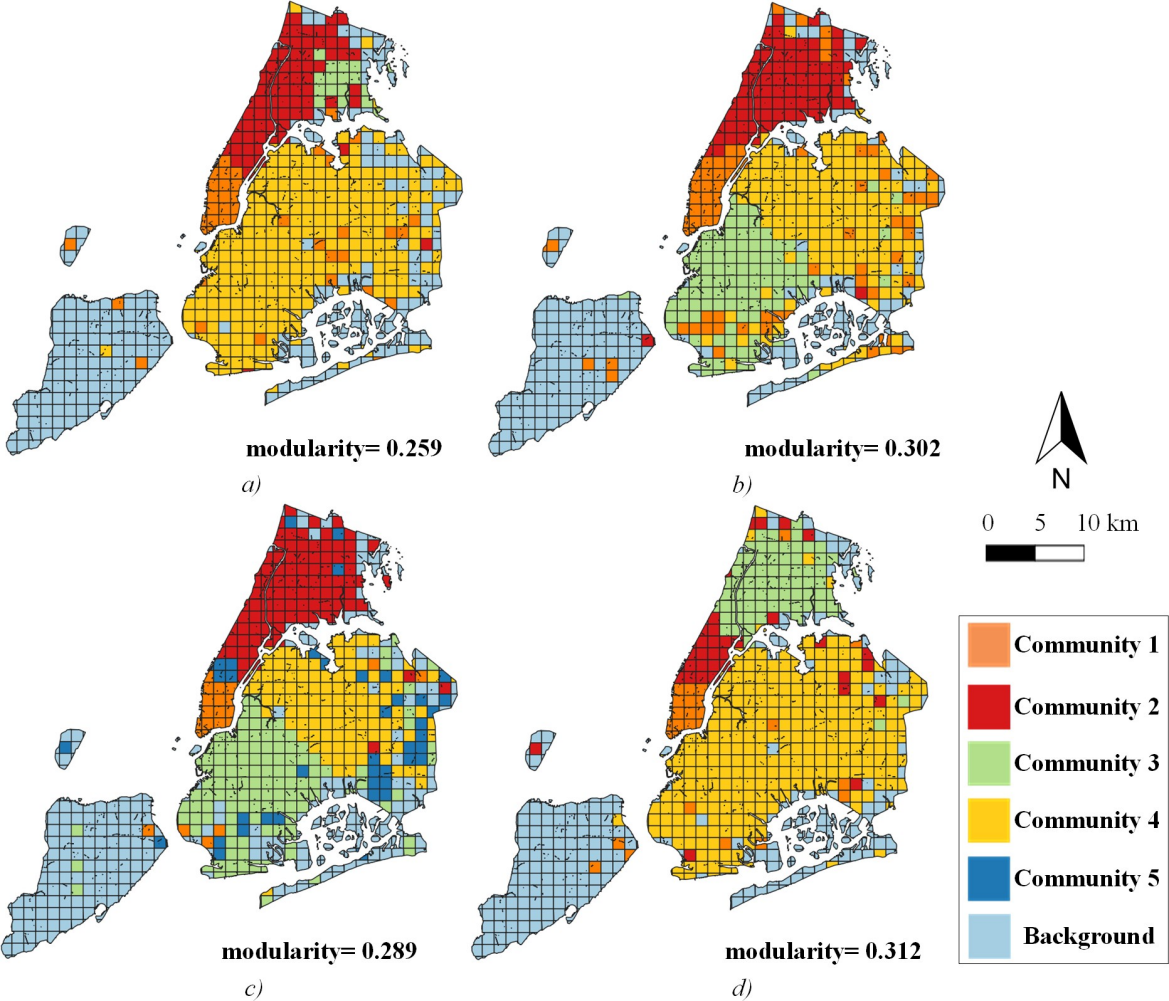
Traffic network community structures. a) *peak1*; b) *peak2*; c) *peak3*; d) *peak4*.

Fig 10 shows the community structures (the background of the network means no trip connected) during each peak period. The OD pairs within a community are densely communicated via taxi flow. Specifically, south Manhattan always forms a separate community, whereas Brooklyn and Queens are sometimes connected. During *peak3*, there is a special community that is geographically dispersed, probably because the connection between transportation hubs that fall into TZs is closer than in other periods (Fig 10C). In general, the community layout is similar to the boroughs distribution of NYC, where areas separated by the sea are often divided into different communities.

Information on nodes and weights of communities is shown in Table 3. Due to the idea of the Fast unfolding algorithm, the communities often have the largest edge weights within the community and are weakly connected to other communities.

**Table 3:** Nodes and weights information of communities.

**Period**	***C***_***k***_	**1**	**2**	**3**	**4**		**∑**_***k***_ ***w***_***ij***_
***Peak1***	1	35993	20440	14	3282		59729
	2	-	20609	73	1021		21703
	3	-	-	93	5		98
	4	-	-	-	4196		4196
	nodes	44	74	17	250		85726
***Peak2***	***C***_***k***_	**1**	**2**	**3**	**4**		**∑**_***k***_ ***w***_***ij***_
	1	70338	13733	2980	2667		89718
	2	-	8489	87	379		8955
	3	-	-	3706	473		4179
	4	-	-	-	3177		3177
	nodes	76	82	103	156		106029
***Peak3***	***C***_***k***_	**1**	**2**	**3**	**4**	**5**	**∑**_***k***_ ***w***_***ij***_
	1	22031	7686	1599	358	15250	46924
	2	-	17639	297	441	12094	30471
	3	-	-	3813	298	890	5001
	4	-	-	-	2389	684	3073
	5	-	-	-	-	8070	8070
	nodes	24	94	126	108	41	93539
***Peak4***	***C***_***k***_	**1**	**2**	**3**	**4**		**∑**_***k***_ ***w***_***ij***_
	1	25796	24281	394	3846		54317
	2	-	34185	3216	3330		40731
	3	-	-	2236	291		2527
	4	-	-	-	11304		11304
	nodes	27	36	72	273		108879

Based on traffic communities, we reveal the weighted degree distribution characteristics of each community. We focus on the weighted degree of the network because it can better reflect the travel behavior of individuals in the urban transportation system rather than the degree of TZs. Given the small number of nodes and the large weighted degree discrepancy of communities, we set a numerical interval *I* for statistics of each community.

**Fig 11:**
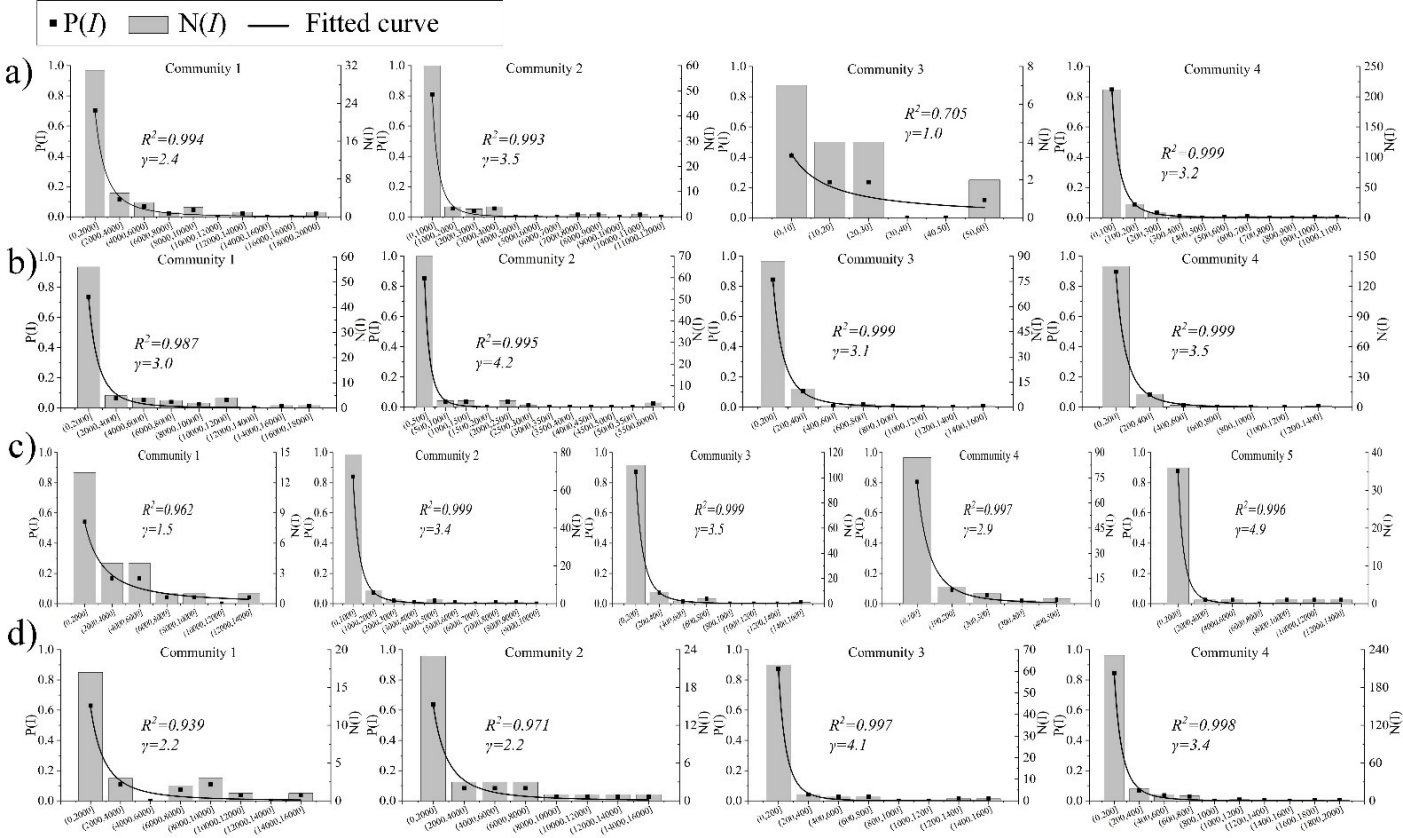
TZs weighted degree distribution. a) *peak1*; b) *peak2*; c) *peak3*; d) *peak4*.

According to the analytical snapshot (Fig 11), the weighted degree of a few TZs is much larger than that of the majority TZs. Furthermore, their weighted degree follows the power-law distribution from a community perspective, that is, *P*(*I*)~*I*^−*γ*^. The R-square of each fitted curve exceeds 0.9 (except community3 in *peak1*), which indicates every community during the rush hours has at least one TZ that has a strong impact on passenger attraction or departure. This is often a precondition for the formation of a tightly connected transport community.

**Fig 12:**
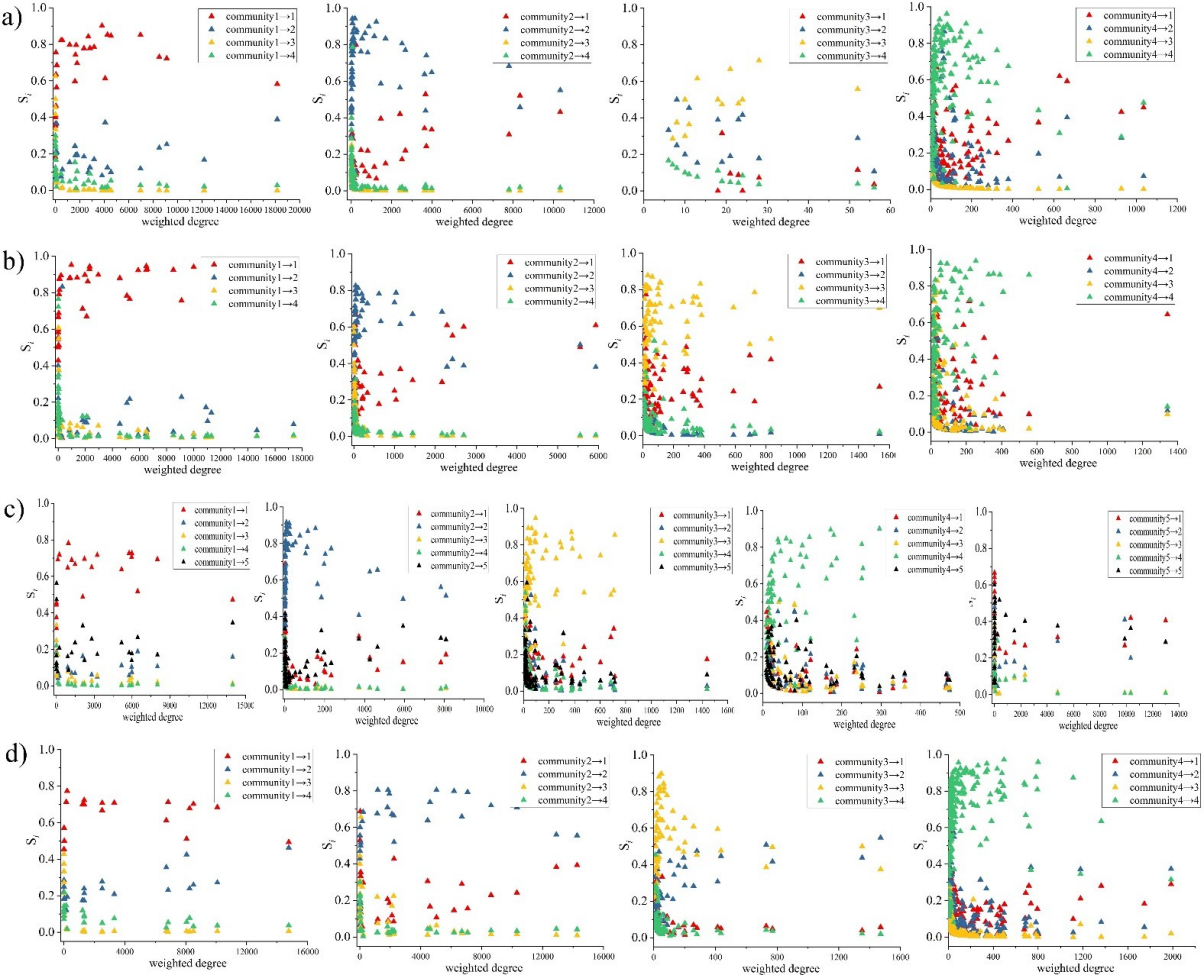
TZs interaction intensity between communities. a) *peak1*; b) *peak2*; c) *peak3*; d) *peak4*.

Fig 12 shows the results of the interactive features of communities in each peak period. The abscissa represents the weighted degree of nodes located in the community, and the ordinate is the interaction intensity index. Each row represents a period, while a subgraph in a row represents the interaction intensity between nodes in a community and other communities during this period. For example, the first subgraph in Fig 12A means the relationship between the weighted degree of nodes in community 1 and the interaction intensity between communities 1 ~ 4 during *peak1*, which is marked with triangles of different colors. Under the overall observation, we can see that as the weighted degree of nodes increases, their interaction intensity within the community always tends to increase, especially when the weighted degree of nodes is situated before the median current. Furthermore, as the weighted degree increases to a certain extent, the intensity of the interaction stabilizes or decreases. It can be understood that the extremely significant or insignificant nodes in most communities often have limited contact with the community in which they are located, while some moderately important nodes have the highest correlation within the community. On the contrary, as the weighted degree of nodes increases, their interaction intensity with other communities always tends to decline but to increase when great to some extent (Fig 12, subgraph 1, 2, 4 of a), subgraph 2, 3, 4 of b), subgraph 1, 2, 3, 5 of c), d)).

Even if most nodes show the above characteristics, we also find a few particular cases, such as subgraph 3 of a), subgraph 1 of b), and subgraph 4 of c), Fig 12. As the weighted degree of nodes increases, the interaction intensity with the community generally continues to increase, while with other communities continues to decline. This interaction feature is the potential expression of the close relationship between nodes within the community. According to the idea of the Fast unfolding algorithm, the increase of modularity is often reflected in the high connection of nodes within the community and the sparse connection outside. Therefore, this is expected to lead to a better division of the community.

Table 4 shows the similarity results for OD pairs during each peak period. The similarity between communities in each peak period is over 50%. It is worth mentioning that the similarity between *peak3* and *peak4* is slightly higher than in other periods because people’s travel purposes are more similar in these two periods, that is, people are more willing to participate in entertainment and social activities on weekends. Overall, traffic community structures are relatively stable over different rush hours.

**Table 4:** Community structures similarity.

Period	*peak1*	*peak2*	*peak3*	*peak4*
***peak1***	1	0.521	0.531	0.532
***peak2***	-	1	0.545	0.524
***peak3***	-	-	1	0.571
***peak4***	-	-	-	1

## 5 Discussion

### 5.1 Spatial community structure of taxi trips

Community detection through travel behavior is an effective strategy to discover the underlying structure of cities [1], the formation of communities often signifies the emergence of compact structures in urban areas. Past research ignored the travel flow of people [36] or only stayed on the community structure layout [20]. We quantify the structure characteristics through network theory to fill this gap. As aforementioned, TZs with numerous passengers may have a limited connection with the community in which they are located, but there are a few exceptions that some of them in the community still have close internal connections (Fig 12). The latter is encouraging because the structure of the traffic community at its period fits very well with the travel within the community, which is more conducive to the formation of compact development urban areas. Some studies have found that the city’s polycentric [37, 38] and compact development [39] is an effective strategy for reducing traffic congestion. Diverse land use in areas ensures that people travel shorter distances and no longer congregate in specific areas of the city [39]. Through the interaction index in communities, we can quickly capture the level of regional interaction of travel flow in key locations and provide some practical insights for the development of compact cities and alleviation of traffic congestion. On the contrary, as the importance of TZs increases, most TZs’ interaction intensity with other communities decreases rapidly until it approaches zero (Fig 12). This is because the modularity changes in the Fast unfolding algorithm are often insensitive to OD pairs with small edge weights. It also indicates that most people prefer to travel within the communities.

A well-divided traffic community often has very little external contact. However, the boundary between community 1 and community 2 is often not very clear, that is, the TZs of high importance in community 1 also undertake most of the travel in community 2 and vice versa. According to Fig 10, community 1 is located in southern Manhattan, while community 2 is located in northern and central Manhattan, even in the Bronx. They are very close and without large geographical separation. Moreover, it can be seen that most of the trips in NYC take place inside Manhattan according to Figs 6 and 7. Although they finally meet the algorithm termination in the community dividing process, it is also the main factor that inhibits the increase in modularity. Sun et al. illustrated that the smaller the modularity, the more congested the traffic network [40]. Therefore, in the actual planning of urban traffic and the compact design of the city, we need to focus on this traffic characteristic. It prevents the better division of communities, likely due to the lack of a specific land-use function within the community. The unique land-use function simultaneously attracts trips between multiple communities, leading to a large gathering of vehicles and people.

### 5.2 Relationship among hotspots, communities and their characteristics

The hotspots of a city can be reflected by the traffic demand, and they globally reveal the critical areas of the entire city. In NYC, they present a cluster distribution layout (Figs 8 and 9). Through the global and local Moran’s I, we can discover the statistically significant autocorrelation of traffic hotspots and find the potential geographical location. The presented results are highly consistent with the hotspots found by KDE, which validates the approach in spatial analysis from the taxi mobility network.

Even if there is only one acknowledged central area in NYC (Manhattan), taxi trips at different periods still form an obvious community structure in space (Fig 10). They are relatively stable over time, with nodes within a community generally adjacent geographically. According to the analysis of this study, hotspots in urban areas are often a requirement for the formation of traffic communities (Fig 11). While their interaction within the community is limited, it is more likely to prevent the formation of a better community structure. As for a polycentric and compact urban development, it seems the best approach is to disperse the hotspots and integrate various land functions.

### 5.3 Potential applications of the framework

Taxi companies may find that the framework of this study is valuable for the identification and prediction of where passengers depart and arrive. It mainly reveals two kinds of information: First, hotspots detection finds the specific location of passengers to ride in different rush hours and identifies the travel destination of people. Capturing the hotspots for passengers in time can provide considerable benefits to taxi companies. Second, the spatial statistical methods reveal the areas where the traffic demand continues to be concentrated, as well as the geographical boundaries with unpopular districts. These hotspots should be given higher priority during the taxi passenger search period.

Urban planners can also get helpful insights from the results. People tend to travel for short distances, so community structure may be formed at any period, while is not ideal enough sometimes. According to the analysis of this study, the reason for preventing a well-division of a community is that some important TZs attract a large number of passengers from other communities, which seems to be due to a lack of required land functions in other communities. Moreover, important TZs can be more decisive in the formation of a community. As a result, a compact and polycentric urban development seems to disperse these key TZs geographically and is equipped with perfect urban functions to alleviate the traffic congestion caused by passenger gathering. The interaction intensity can be used to assess the level of compaction of a city. For example, urban planners can capture the key areas that inhibit the compact development in the city by judging their interaction intensity between the communities and plan for better urban areas by exploiting the land use functions.

## 6 Conclusion

This study proposed an analytical framework aims at revealing the travel hotspots, their spatial autocorrelation, and the community structure characteristics through the graph-based approach and complex network theory. The main conclusions are as follows.

1. Penn Station and Grand Central Terminal are hotspots for taxis at any peak times of the day. When traveling for commuting, the destinations for passengers in the morning rush hours are concentrated in many financial and commercial areas of Manhattan, while are widely distributed in more diverse places in the evening. When traveling for entertainment, the hotspots for taxi rides during the daytime are concentrated in large department stores, parks, and churches, whereas people are more willing to arrive at theaters at night. Travel demand in NYC shows a strong positive spatial autocorrelation during multiple peak times. In the meanwhile, taxis are more inclined to carry passengers in central Manhattan and transport passengers to peripheral areas.
2. Over different rush hours, the movement of taxis will form a relatively stable, geographically clustered community structure. After empirical analysis of NYC through weighted distribution and interaction intensity, we found several interesting phenomena: a) Every community exists a very small number of TZs are undertake a large amount of passenger travel activities. Moreover, if the numerical interval is used for statistics, the weighted degree of TZs in the community follows the power-law distribution. b) As the weighted degree of TZs increases, the interaction intensity with the community where they are located first strengthens, then stabilizes, and finally weakens, which is probably due to the lack of land use functions in other neighboring communities. In other words, the formation of a community is determined by the key TZs with numerous passengers, but these TZs may have limited connection with the community in which they are located.

Although taxis can reflect residents’ travel behavior, there is an inherent sampling deviation, because not all urban residents choose taxis as their travel mode. Future work will improve this problem and extend the analysis to a longer time span to understand the spatiotemporal pattern and the mechanism of the community structure evolution. It can also be further studied in combination with land use data to obtain more refined results.
